# Tensile Strength and Electromagnetic Wave Absorption Properties of B-Doped SiC Nanowire/Silicone Composites

**DOI:** 10.3390/nano15171298

**Published:** 2025-08-22

**Authors:** Yiwei Wang, Qin Qin, Jingyue Chen, Xiang Lu, Jialu Yin, Ranhao Liu, Peijie Jiang, Jianlei Kuang, Wenbin Cao

**Affiliations:** Department of Inorganic Nonmetallic Materials, School of Material Science and Engineering, University of Science and Technology Beijing, Beijing 100083, China; m202310369@xs.ustb.edu.cn (Y.W.); qinqin202020@163.com (Q.Q.); 15620174889@163.com (J.C.); m202310339@xs.ustb.edu.cn (X.L.); m202320548@xs.ustb.edu.cn (J.Y.); m202420483@xs.ustb.edu.cn (R.L.); m202410305@xs.ustb.edu.cn (P.J.)

**Keywords:** SiC nanowires, silicone matrix composites, electromagnetic-wave-absorbing material, mechanical property enhancement

## Abstract

To investigate the synthesis route and electromagnetic wave absorption performance of SiC nanowires (SiC-NWs), boron was simultaneously employed as both a catalyst and a dopant, and the doped nanowires were embedded into a silicone matrix to fabricate SiC-NW/silicone composites with enhanced mechanical properties and microwave attenuation. Boric acid significantly increased the yield of SiC-NWs, while boron doping enhanced both conductive and relaxation losses. The subsequent nanowire pull-out mechanism improved the tensile strength of the composites by 185%, reaching 5.7 MPa at a filler loading of 5 wt%. The three-dimensional SiC-NW network provided synergistic dielectric and conductive losses, along with good impedance matching, achieving a minimum reflection loss of −35 dB at a thickness of 3.5 mm and an effective absorption bandwidth of 4.2 GHz within the 8.2–12.4 GHz range, with a nanowire content of only 5 wt%.

## 1. Introduction

The rapid development of 5G/6G communication networks, automotive and airborne millimeter-wave radars, low-orbit satellites, unmanned aerial vehicles, and wearable/portable electronics has intensified the challenges of electromagnetic (EM) interference and radiation protection. In practical applications, EM absorbers are widely employed to reduce radar cross-section on conformal coatings, suppress crosstalk and spurious emissions in densely integrated electronic modules, and protect sensitive devices and human operators from EM radiation during long-term service. These application scenarios impose stringent requirements: absorbers should be lightweight and thin, exhibit sufficient mechanical compliance to conform to complex curved surfaces, maintain stable performance under environmental stresses such as thermal-humidity cycling, and allow scalable, large-area processing [1,2]. Conventional absorbers—magnetic metals, carbon-based materials, and conductive polymers—although demonstrating excellent attenuation capability in specific frequency bands, generally suffer from high density and susceptibility to oxidation/corrosion, or require high filler loadings to maintain performance, leading to degradation in mechanical properties and processability [3,4,5]. Therefore, there is an urgent demand for flexible EM absorbers that can maintain high attenuation efficiency and structural integrity under harsh service conditions [6,7,8].

Combining lossy fillers with flexible polymer matrices is an effective strategy to meet these requirements, as impedance matching can be optimized to balance absorption performance and mechanical flexibility. For example, Shi et al. [9] incorporated polypyrrole-coated carbon nanofibers into silicone rubber textiles, achieving a minimum reflection loss (*RL*_min_) of ~−25 dB and an effective absorption bandwidth (*EAB*) of 4.2 GHz at a 10 wt% loading and 4.0 mm thickness; Zhang et al. [10] embedded MoO_2_/N-doped carbon hetero-nanowires into a TPU matrix, obtaining *RL*_min_ ≈ −35 dB and *EAB* ≈ 3.26 GHz at a high loading of 40 wt% with 2.3 mm thickness; Li et al. [11] prepared Fe–TiN fiber/PDMS composites with *RL*_min_ ≈ −20 dB and *EAB* ≈ 12.2 GHz at only 15 wt% loading and 1.8 mm thickness; Li et al. [12] developed Ti-HEO/ACET composites achieving *RL*_min_ ≈ −52.3 dB and *EAB* ≈ 6.12 GHz at 17.5 wt% loading and 2.03 mm thickness. While these studies highlight the great potential of flexible composite absorbers, they also mention common limitations: filler loadings increase density and processing viscosity; 2D carbon or metallic fillers are prone to oxidation; ideal impedance matching often requires substantial thickness, limiting ultrathin applications; in addition, some high-performance designs rely on complex multistep fabrication processes that hinder large-scale deployment.

Among various fillers, SiC nanowires stand out due to their one-dimensional morphology and high aspect ratio, which facilitate the construction of conductive-polarization networks [13], while intrinsic structural defects (e.g., stacking faults, twins) enhance interfacial polarization and dielectric loss [14,15,16,17], making them ideal dielectric-type absorbers [18,19,20,21,22,23]. Furthermore, SiC possesses chemical inertness, thermal stability, oxidation resistance, and low density, enabling stable performance under high-temperature and corrosive environments [24,25,26,27]. The scalable production of high-quality SiC nanowires is a key step for practical applications [28]. Compared with chemical vapor deposition (CVD), polymer pyrolysis, and template-assisted growth, catalyst-assisted carbothermal reduction offers advantages such as low-cost precursors, lower reaction temperatures, shorter growth cycles, and higher yields, along with the optimization of nanowire morphology and defect structures to enhance dielectric properties [29,30,31,32,33]. Silicone rubber, as a matrix, offers wide-temperature elasticity, excellent hydrophobicity and weather resistance, and good electrical insulation, enabling conformal adhesion to complex curved metallic surfaces without cracking or loss of flexibility. Its favorable processability allows the fabrication of thin and uniform absorber layers, outperforming brittle matrices such as epoxy resin or polyimide in mechanical compliance [34,35].

In this work, we propose a flexible, all-dielectric EM absorber by incorporating SiC nanowires into a silicone rubber matrix. The SiC nanowires are synthesized via a boric-acid-catalyzed carbothermal reduction method, in which boron plays a dual role as both a growth catalyst and a doping element [36]. As a catalyst, boron promotes the rapid growth of high-aspect-ratio B-doped nanowires with low stacking-fault density, facilitating the formation of continuous 3D conductive networks and ensuring mechanical integrity. As a dopant, boron is substitutionally incorporated into the SiC lattice, increasing carrier concentration and introducing defect and interfacial polarization centers, thereby enhancing both conductive and relaxation loss mechanisms. Through the synergistic effects of morphology optimization and dielectric-loss modulation, together with thickness optimization (approaching the quarter-wavelength condition in the X-band), the composite achieves *RL*_min_ ≈ −35 dB and *EAB* ≈ 4.2 GHz at only 5 wt% filler loading, while enhancing tensile strength by ~185%. This design effectively addresses the dual challenge of mechanical reinforcement and EM attenuation in flexible all-dielectric absorbers and provides a feasible pathway toward scalable, conformal absorber films for 5G/6G platform applications.

## 2. Materials and Methods

### 2.1. Preparation of B-Doped SiC Nanowires

A silica sol/activated-carbon slurry was first prepared at a molar ratio of Si:C = 1:4. Sodium hexametaphosphate (SHMP, Sinopharm Chemical Reagent Co., Ltd, Shanghai, China) was added as a dispersant. Then, the mixture was vigorously stirred at 1000 rpm for 2 h. After drying, boric acid powders (Sinopharm Chemical Reagent Co., Ltd, Shanghai, China) were introduced at a molar ratio of B:C = 0:4 (blank, denoted 0B–SiC), 0.01:4 (0.01B–SiC), 0.025:4 (0.025B–SiC), and 0.05:4 (0.05B–SiC), respectively. The dried powders were mixed by ball-milling at 100 rpm for 4h. The resulting precursors were loaded into crucibles and covered with a graphite sheet. Carbothermal reduction was carried out in a multimode-cavity microwave furnace under flowing Ar (0.25 L min^−1^). The temperature was ramped to 1400 °C and held for 30 min. SiC nanowires grew on the underside of the graphite sheet. The product was calcined in a muffle furnace at 700 °C for 2 h, after which the graphite sheet was removed. A second calcination was then carried out at 650 °C for 2 h, ultimately yielding carbon-free B-doped SiC nanowires.

### 2.2. Preparation of B–SiC Nanowire–Silicone Composites

To fabricate the composites, silicone resin components A and B (Dongguan Hongcheng Silicone Technology Co., Ltd., Dongguan, China) and the SiC nanowires were weighed in a mass ratio of 50:50:x (x = 0, 1, 3, 5, 10). Component A and the nanowires were first thoroughly blended by mechanical stirring, during which a small amount of volatile silicone oil (Dowcorning, Shanghai, China) was added. After that, part B was added, and stirring continued until a homogeneous mixture was obtained. The mixture was degassed under vacuum to 0.02 MPa, poured into a mold, and cured at 100 °C for 30 min to form the silicone/B–SiC nanowire composites.

### 2.3. Characterizations and Calculation

Phase composition, microstructure, and mechanical performance were characterized as follows. X-ray diffraction (XRD, Bruker D8, Karlsruhe, Germany) was used to identify crystalline phases. Raman spectra were collected with a Reflex spectrometer (Gloucestershire, UK). Microstructural observations were carried out using field-emission scanning electron microscopy (FE-SEM, ZEISS Ultra 55, Oberkochen, Germany) and high-resolution transmission electron microscopy (HRTEM, JEOL JEM-2100F, JEOL., Tokyo, Japan). The tensile properties of the resulting composites were measured on an electronic universal testing machine (KY-D4504, Tianjin Yiwei Jingrui Trading Co., Ltd., Tianjin, China). The complex permittivity and permeability (*ε*’ and *μ*’) of the composites in the 8.2–12.4 GHz band were obtained with a vector network analyzer (Agilent N5230A, Agilent Technologies, Santa Clara, CA, USA). Reflection loss (*RL*) values were then calculated from the measured parameters using the standard transmission-line Equations (1) and (2).

(1) $$Z_{\mathrm{in}}=Z_{0}\sqrt{\frac{\mu_{r}}{\varepsilon_{r}}}\tanh(j\frac{2\pi fd}{c}\sqrt{\mu_{r}\varepsilon_{r}})$$

(2) $$RL=20\log_{10}\left|\frac{Z_{\mathrm{in}}-Z_{0}}{Z_{\mathrm{in}}+Z_{0}}\right|$$

*Z*_0_ and *Z*_in_ represent the free-space impedance and the input impedance of the absorber, respectively; *ε_r_* and *μ_r_* are the absorber’s complex relative permittivity and permeability; π is the mathematical constant pi; *f* is the frequency of the incident microwave; *d* is the absorber thickness; and *c* is the speed of light in a vacuum.

## 3. Results and Discussion

**Figure 1:**
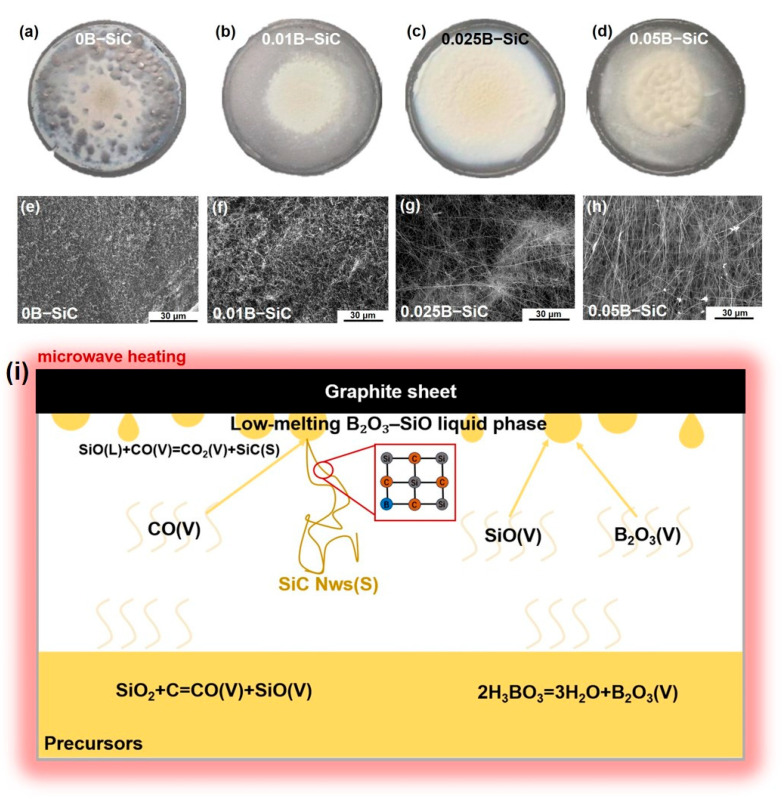
(**a**–**d**) Macro photographs and (**e**–**h**) SEM images of as-synthesized SiC nanowires with varying B_2_O_3_ contents. (**i**) Schematic illustration of the B_2_O_3_-catalyzed VLS growth mechanism of B-doped SiC nanowires.

Figure 1 shows the macroscopic photographs (Figure 1a–d) and SEM micrographs (Figure 1e–h) of SiC nanowires obtained with increasing boron additions and a schematic illustration of the B_2_O_3_-catalyzed VLS growth mechanism of B-doped SiC nanowires. In the macroscopic images, the 0.025B–SiC sample shows the highest nanowire yield and the most uniform growth. The other three samples exhibit lower yields or marked nonuniformity. All four SEM images confirm the formation of SiC nanowires. However, when only a small amount of boric acid is introduced, the nanowires are short and possess low aspect ratios, making it impossible to establish an effective conductive network. As the boric-acid content increases, nanowire length rises sharply: for both 0.025B–SiC and 0.05B–SiC, the average length exceeds 200 μm. But the macroscopic inhomogeneity observed in the 0.05B–SiC batch makes 0.025B–SiC the optimum catalyst loading.

Under high-temperature conditions, the SiO_2_–C precursors undergo a gas-phase reaction, generating SiO and CO vapors according to Equation (3). As the partial pressures of these vapors increase, SiO and CO diffuse to the surface of the carbon paper, where they nucleate and grow into nanowires via Equation (4), following the classical vapor–solid (VS) growth mechanism. At this stage, nanowire formation is purely driven by the condensation of gaseous species on the solid surface, without any intermediate liquid phase. In the absence of boric acid, growth proceeds mainly through this VS pathway, with the nucleation density and wire morphology primarily determined by vapor supersaturation and surface conditions, resulting in nanowires with low aspect ratios and poor morphology.

(3) $${\mathrm{SiO}}_{2}+C=\mathrm{SiO}+\mathrm{CO}$$

(4) $$\mathrm{SiO}+3\mathrm{CO}={\mathrm{SiC}+2\mathrm{CO}}_{2}$$

**Figure 2:**
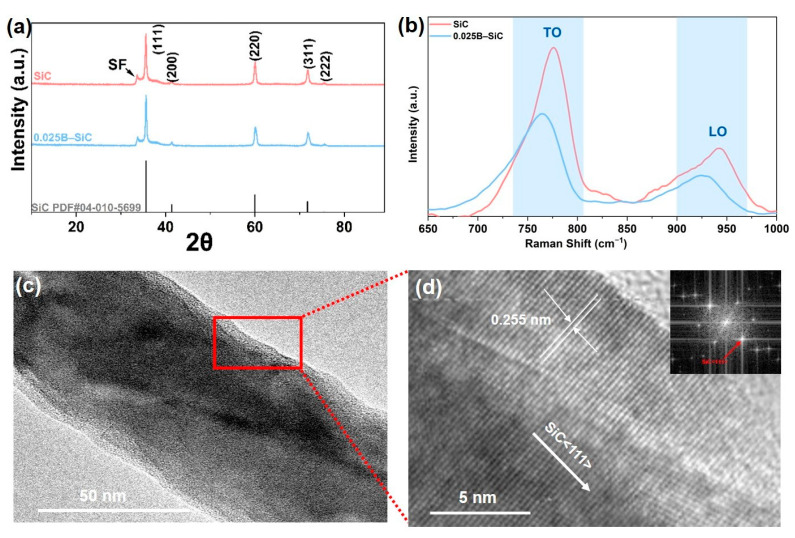
(**a**) XRD patterns and (**b**) Raman spectra of 0B–SiC and 0.025B–SiC; (**c**,**d**) TEM and HRTEM images of 0.025B–SiC nanowires.

When boric acid (H_3_BO_3_) is introduced, the growth mode of SiC nanowires shifts from the vapor–solid (VS) route to a boric-acid-catalyzed vapor–liquid–solid (VLS) route, as illustrated in Figure 1i. Upon heating, H_3_BO_3_ first dehydrates to metaboric acid (HBO_2_) and then further dehydrates to boron oxide (B_2_O_3_). A portion of the resulting B_2_O_3_ evaporates and condenses on the surface of the carbon paper to form nanoscale droplets, while the remainder reacts with SiO vapor to produce a low-melting B_2_O_3_–SiO liquid phase. These catalytic droplets act as nucleation centers, significantly enhancing interparticle and interfacial diffusion, lowering the synthesis temperature, and accelerating nanowire growth. However, excessive H_3_BO_3_ introduces too much liquid, leading to droplet coalescence and gravitational flow, which creates spatial gradients in nucleation density and impairs both the macroscopic uniformity and microscopic consistency of the nanowires. In addition to acting as VLS catalysts, boron atoms in the liquid phase can diffuse into the SiC lattice during crystal growth, partially substituting for silicon atoms. This lattice incorporation alters the crystal parameters, thereby affecting the morphology and yield of the nanowires. Moreover, after the introduction of boric acid, a small fraction of boron enters the lattice to replace Si atoms, further changing the lattice parameters. Figure 2a,b compare the X-ray diffraction (XRD) and Raman spectra of 0B–SiC nanowires and 0.025B–SiC. All diffraction peaks match the standard pattern of cubic SiC (JCPDF: 29-1129). In the boric-acid-added sample, the main SiC (111) diffraction peak shifts toward a higher 2θ value, confirming that boric acid functions not only as a catalyst but also introduces a fraction of boron atoms into the lattice, substituting for silicon atoms and thereby reducing the lattice parameter. Moreover, both the undoped and B-doped samples exhibit a small satellite peak adjacent to the main (111) reflection. This feature originates from stacking faults (SFs) introduced during nanowire growth. The SF density index, *d*_SF_, was estimated with Equation (5):

(5) $$d_{\mathrm{SF}}=I_{\mathrm{SF}}/I_{\left(200\right)}$$

in which *d*_SF_ is the intensity of the SF peak, and *I_(_*_200)_ is the intensity of the (200) diffraction peak. The calculated values are *d*_SF_ = 2.47 for undoped SiC nanowires, and *d*_SF_ = 2.09 for B-doped SiC nanowires, demonstrating that boron incorporation markedly lowers the SF density.

According to Equations (3) and (4), SiC nanowires grow from the intermediate gaseous species SiO and CO via both vapor–solid (VS) and vapor–liquid–solid (VLS) mechanisms. In the VS route, the formation of stacking faults reduces the energy required for axial growth, leading to a relatively strong SF peak. When boric acid is added, the growth switches to the VLS pathway: the liquid catalyst phase lowers the reaction barrier directly, so the process no longer relies on stacking faults, resulting in a lower SF density.

The Raman spectra also prove boron incorporation. Both samples show the characteristic transverse-optical (TO, ≈796 cm^−1^) and longitudinal-optical (LO, ≈972 cm^−1^) modes of SiC. But in 0.025B–SiC, the TO mode is red-shifted and markedly broadened, while the LO mode is also red-shifted, and its intensity relative to the TO mode is diminished. These changes suggest that B doping introduces free holes and point defects, with the associated strain altering the phonon spectrum. This also confirms that boron is incorporated into the SiC lattice instead of merely serving as the catalyst for the VLS reaction.

Figure 2c,d present a transmission electron microscope (TEM) overview and the corresponding high-resolution TEM image of a 0.025B–SiC nanowire. The measured lattice fringe spacing of 0.255 nm agrees with the (111) planes of SiC. The selected-area electron diffraction pattern exhibits sharp spots characteristic of a single-crystal SiC nanowire growing along the (111) direction.

**Figure 3:**
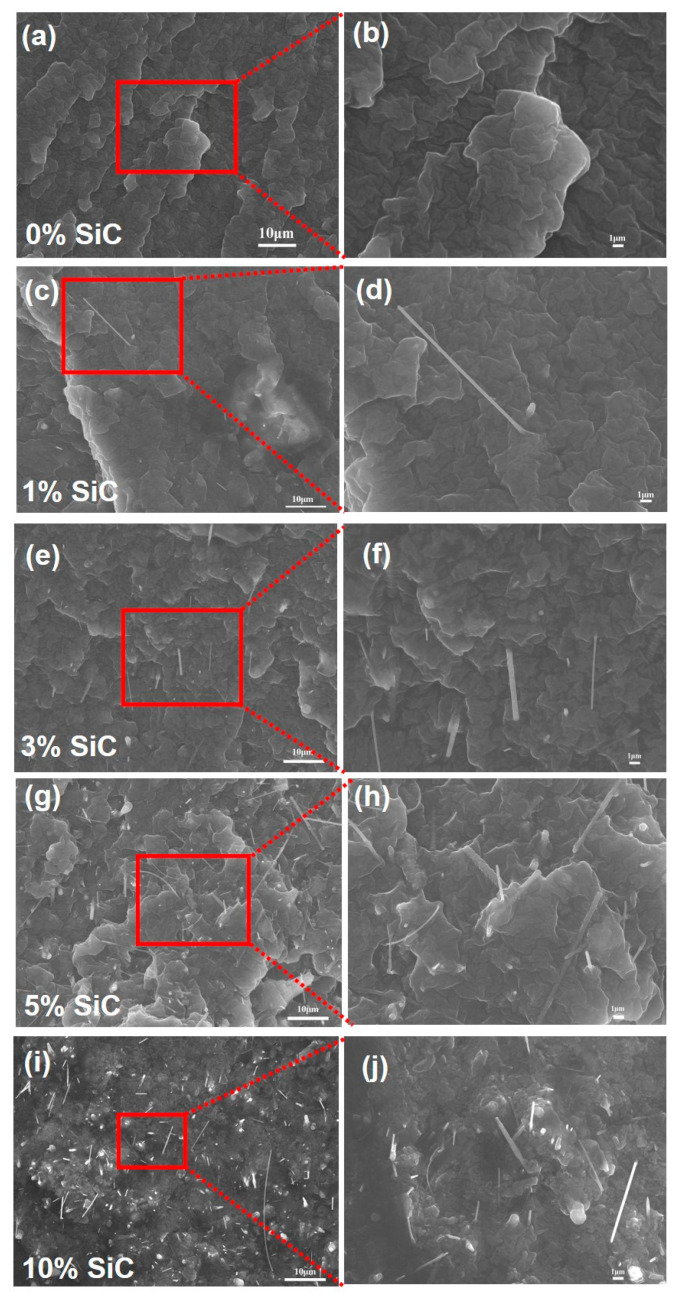
Cross-sectional SEM images of B-doped SiC nanowire/silicone composites with different SiC loadings: (**a**,**b**) 0 wt%, (**c**,**d**) 1 wt%, (**e**,**f**) 3 wt%, (**g**,**h**) 5 wt%, and (**i**,**j**) 10 wt%. Images (**a**,**c**,**e**,**g**,**i**): 1500×; images (**b**,**d**,**f**,**h**,**j**): 4000×.

Figure 3 presents cross-sectional SEM micrographs of the composites prepared with various SiC nanowire loadings. After embedding 0.025B–SiC nanowires in the silicone matrix, the fracture-surface SEM images reveal the mechanism responsible for the composite’s mechanical reinforcement. As the SiC nanowire content rises from 0.25 wt% to 5 wt%, the fracture surface evolves from a smooth, featureless tear to a layered, rough morphology containing microvoids and pulled-out fibers. This transition implies that toughening is governed by a synergistic interplay between interfacial load transfer and matrix strain-hardening, rather than by brittle fiber pull-out alone [37]. During tensile loading, SiC nanowires that are well-bonded to the silicone matrix first share the external stress through interfacial shear, effectively lowering local stress concentrations within the composite. Once debonding initiates, the nanowires slide under friction while the surrounding silicone undergoes elastoplastic deformation, dissipating energy [38]. Nanowires that remain partially embedded bridge the ensuing cavities and exert crack-closing stresses, thereby retarding crack propagation. At 10 wt% loading, however, agglomeration and entanglement hinder uniform stress transfer across the now abundant internal interfaces, resulting in a slight decline in mechanical performance.

**Figure 4:**
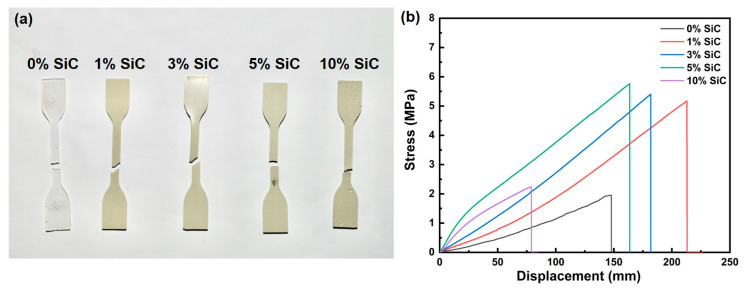
(**a**) Photographs of tensile specimens prepared from SiC nanowire/silicone composites; (**b**) tensile stress–strain data for the corresponding composites.

Figure 4 summarizes the tensile performance of the SiC nanowire/silicone composites as a function of filler loading. In the fracture photographs (Figure 4a), every specimen fails within the gauge section, indicating that the fracture originates in the region of uniform stress. The corresponding stress–strain curves (Figure 4b) confirm that introducing SiC nanowires substantially reinforces the otherwise weak silicone matrix. The neat polymer exhibits an ultimate tensile strength of only 2 MPa, whereas composites containing 1, 3, 5, and 10 wt% nanowires reach 5.2, 5.4, 5.7, and 2.2 MPa, respectively—corresponding to strength enhancements of 160%, 170%, 185%, and 10% relative to the unreinforced matrix. The improvement through 5 wt% is consistent with the well-dispersed nanowire network observed in Figure 3, which facilitates efficient load transfer across the polymer-filler interface and allows distributed energy dissipation. This reinforcing effect mainly originates from the high aspect ratio and strong interfacial adhesion of SiC nanowires, which enable them to act as effective stress-bridging elements. During tensile deformation, well-bonded nanowires can transfer external stress to the matrix by shear, while their pull-out and sliding processes absorb and dissipate additional mechanical energy, delaying crack propagation. Furthermore, the entangled three-dimensional nanowire network forms a percolated skeleton inside the silicone, which restricts local deformation of the soft matrix and contributes to strain-hardening. The accompanying fracture displacement follows the same trend: it grows from ~60 mm in the neat silicone to >150 mm at 1–3 wt% and reaches ~200 mm at 5 wt%, reflecting the ability of the interconnected nanowire framework to bridge microcracks and delay catastrophic failure. At 10 wt % loading, excessive nanowires not only agglomerate but also introduce microvoids and weak interfaces that break up the continuous reinforcement path and create local stress concentrators; these defects limit efficient load transfer, and nanowire agglomeration introduces stress concentration, leading to matrix embrittlement, so the tensile strength (~2.2 MPa) and maximum displacement (<100 mm) both decline—behavior that matches the microstructural features observed in Figure 3.

**Figure 5:**
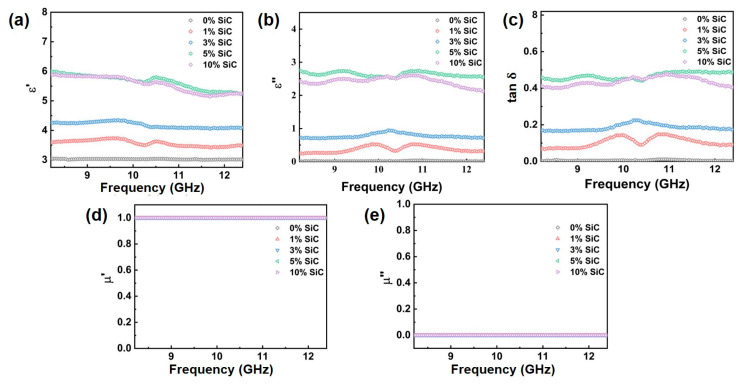
Electromagnetic parameters of B-doped SiC nanowire/silicone composites with different SiC loadings: (**a**) real part of permittivity (*ε*′), (**b**) imaginary part of permittivity (*ε*″), (**c**) dielectric loss tangent (tan δ), (**d**) real part of permeability (*μ*′), and (**e**) imaginary part of permeability (*μ*″).

Figure 5 plots the real (*ε*′) and imaginary part (*ε*″) of the permittivity, dielectric-loss tangent (tan δ) and the real (*μ*′), and imaginary part of the permeability (*μ*″) of the composites as a function of SiC nanowire content. For the silicone without SiC nanowires, the neat matrix displays an almost frequency-independent real permittivity of ≈3, an imaginary part close to zero, and a tan δ below 0.02 across the entire band, confirming its intrinsically insulating nature. When the filler increases from 1 wt% to 5 wt%, all three quantities rise monotonically, reflecting the progressive establishment of a three-dimensional conductive network (3D network). The network can boost both polarization and conduction losses. This increase arises because the interconnected SiC nanowires form abundant interfacial regions with the insulating silicone, which induces Maxwell–Wagner–Sillars polarization, while the higher carrier concentration introduced by boron doping enhances conduction loss. In addition, the elongated one-dimensional nanowires act as efficient pathways for charge transport, allowing oscillating charges to move under the microwave field and dissipate energy as Joule heat. Above 5 wt%, the curves flatten: neither *ε*′ nor *ε*″ nor tan δ shows a further appreciable change at 10 wt%. At such high filler content, agglomeration of nanowires reduces the effective surface area available for interfacial polarization, while excessive conductivity leads to impedance mismatch with free space, so further increases in dielectric response are suppressed. This explains why the permittivity parameters no longer rise despite higher filler addition.

This behavior can be rationalized with the empirical mixing relation proposed by Lichtenecker, which expresses the effective permittivity of a binary composite as

(6) $$e_{\mathrm{ff}}^{K}=V_{A}\varepsilon_{A}^{K}+V_{B}\varepsilon_{B}^{K}$$

where *V*_A_ and *V*_B_ are the volume fractions, and $\varepsilon_{A}^{K}$ and $\varepsilon_{B}^{K}$ are the permittivities of constituents A and B, respectively, while *K* is a dimensionless fitting parameter. For a perfectly homogeneous mixture, *K* approaches zero, and experiments show that Equation (6) is most accurate when the volume fraction of the high-permittivity phase is small. The greater the contrast between *ε*_A_ and *ε*_B_, the narrower the composition range over which the formula holds.

Consequently, in the 1–5 wt% interval, the gradual rise in SiC volume fraction falls within the validity domain of Equation (6). Once the loading reaches 10 wt%, however, the composite lies outside the range where Equation (6) can reliably predict $e_{ff}^{K}$ At this higher content, the interconnected SiC network becomes sufficiently dense to reflect, rather than absorb, a larger share of the incident electromagnetic energy, so *ε*′, *ε*″, and tan δ show no additional increase.

Vector network analyzer measurements revealed that the real part (*μ*′) of the complex permeability for the composites remains close to 1, while the imaginary part (*μ*″) approaches 0 throughout the 8.2–12.4 GHz frequency range. This indicates that the composites exhibit negligible magnetic response and that magnetic loss can be ignored within this band. Such behavior is consistent with the intrinsic non-magnetic nature of both SiC nanowires and the silicone matrix, and the boron dopant does not introduce any ferromagnetic or ferrimagnetic phases. At high frequencies, even materials with weak magnetic moments typically exhibit natural resonance frequencies outside the X-band, leading to vanishing *μ*″ values.

**Figure 6:**
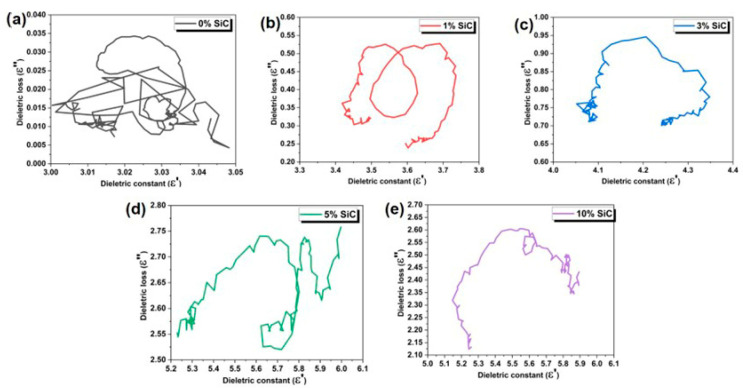
Cole–Cole plots of B-doped SiC nanowire/silicone composites with different SiC loadings: (**a**) 0 wt%, (**b**) 1 wt%, (**c**) 3 wt%, (**d**) 5 wt%, and (**e**) 10 wt%.

Figure 6 compares the Cole–Cole plots of the composites as the SiC nanowire loading increases from 0 to 10 wt%. For the neat silicone (0 wt% SiC), the Cole–Cole plot shows a tiny, irregular cluster centered at *ε*′ ≈ 3.0 with *ε*″ < 0.04 instead of a discernible semicircle. Such a flattened, almost point-like locus indicates that the relaxation strength is too small to form a visible Debye arc within the 8–12 GHz window. The matrix behaves as an essentially ideal insulator whose dipolar polarization is frequency-independent and whose conduction loss is below the detection limit. Introducing 1 wt% SiC (Figure 6b) broadens the loop and shifts it upward, signifying additional interfacial polarization and the first signs of incipient conductive paths, which give a modest rise in dielectric loss. At 3 wt% (Figure 6c), the trajectory flattens and elongates, evidence that a partial conductive network now contributes alongside interfacial polarization; conduction loss becomes appreciable and a wider spread of relaxation times appears. With 5 wt% nanowires (Figure 6d), the curve opens further and moves to *ε*′ ≈ 5.2–6.0 and *ε*″ ≈ 2.5–2.8, showing that the composite is close to the percolation threshold and can dissipate electromagnetic energy efficiently through combined polarization and Joule heating. At 10 wt% (Figure 6e), the loop contracts slightly, and its maximum *ε*″ falls, confirming that excessive nanowire agglomeration reduces effective interfacial area and produces local short-circuits, thereby weakening both polarization and conduction losses.

Overall, increasing the SiC nanowire content from 0 to 5 wt% steadily elevates *ε*′ and *ε*″, while the Cole–Cole plots evolve from an irregular cluster toward increasingly open trajectories that reflect the contribution of dielectric loss and conduction loss. Beyond 5 wt%, the incremental benefit saturates or even declines owing to agglomeration and impedance mismatch, so a filler level of roughly 3–5 wt% strikes the best balance between providing abundant dielectric-loss channels and maintaining good impedance matching with free space.

**Figure 7:**
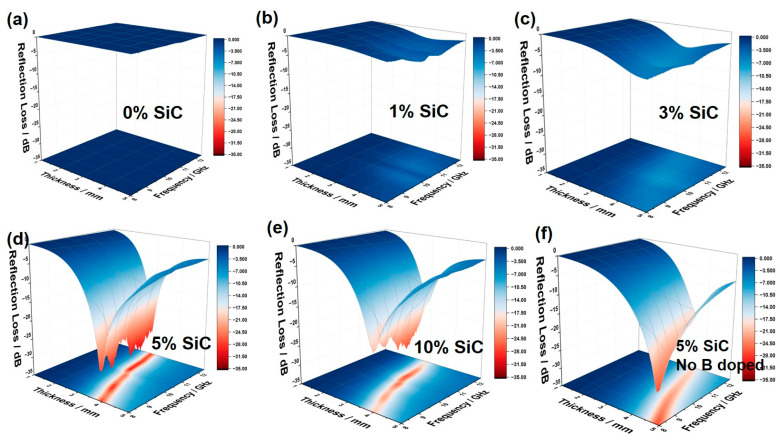
(**a**–**e**) Three-dimensional maps of the calculated reflection loss (*RL*) for SiC nanowire/silicone composites with different SiC loadings (0 wt%, 1 wt%, 3 wt%, 5 wt%, 10 wt%); (**f**) three-dimensional map of the calculated reflection loss for the 5 wt% SiC composite without B doping.

By substituting the measured complex permittivity and permeability into Equations (1) and (2), the theoretical reflection loss (*RL*) of 1–5 mm absorbers was calculated for 8.2–12.4 GHz (Figure 7a–e). In addition, Figure 7f presents the *RL* performance of the composite containing 5 wt% undoped SiC nanowires for comparison. Neat silicone (Figure 7a) shows practically no attenuation (*RL*_min_ ≈ 0 dB) because the polymer is nearly loss-less and non-conductive. This agrees with the Cole–Cole plot and confirms that the matrix is almost transparent to microwaves. Adding 1 wt% SiC nanowires enables measurable absorption, but the minimum *RL* is still above −10 dB, indicating that the modest increase in *ε*′ and *ε*″ produced by limited interfacial polarization remains insufficient. Increasing the loading to 3 wt% (Figure 7c) lowers the minimum *RL* to about −15 dB at 3–4 mm thickness, reflecting a broader distribution of relaxation processes. At 5 wt% (Figure 7d), the performance improves markedly: with 2.5–4 mm thickness the *RL*_min_ approaches −35 dB, and the bandwidth where *RL* ≤ −10 dB extends beyond 8 GHz across 8.2–12.4 GHz. Raising the content to 10 wt% (Figure 7e) still yields minima below −30 dB, but the attenuation band narrows and shifts to lower frequency because the higher *ε*′ and *ε*″ intensify intrinsic loss, while nanowire agglomeration weakens impedance matching and reduces the interfacial-polarization contribution. The optimum absorption is found at a thickness of 3.5–4.0 mm. The effective wavelength of microwaves inside the composite, denoted *λ*_m_, is defined by

(7) $$\lambda_{m}=\frac{c}{f\sqrt{\varepsilon^{\prime }\mu^{\prime }}}$$

where *c* is the speed of light, *f* the center frequency of the absorption band, and *ε*′ and *μ*′ are the real parts of the permittivity and permeability, respectively. Equation (7) shows that 3.5–4 mm corresponds closely to one-quarter of this effective wavelength, so reflections from the front and rear interfaces acquire a π phase difference and cancel, giving near-perfect impedance matching. At the same time, this path length lets microwaves traverse the three-dimensional SiC nanowire network long enough for conductive, dipolar, and interfacial mechanisms to dissipate the transmitted energy, resulting in the deepest *RL* (≈−35 dB) and the broadest effective bandwidth observed.

From the comparison between Figure 7d and Figure 7f, it is evident that, at the same SiC loading (5 wt%), the B-doped composite exhibits significantly higher reflection loss (*RL*) intensity and a broader effective absorption bandwidth (*EAB*) than the undoped sample. This difference primarily originates from the distinct morphology of the nanowires. As shown in Figure 1, the B-doped sample, benefiting from boric-acid-assisted catalysis during growth, develops nanowires with a higher aspect ratio, more uniform spatial distribution, and interwoven network structure, thereby forming a continuous three-dimensional conductive network throughout the composite. Such a network facilitates multiple reflections, scattering, and propagation of electromagnetic waves within the material, markedly improving energy coupling and dissipation efficiency. In contrast, the undoped sample, with its lower aspect ratio, fails to establish continuous conductive pathways between nanowires, resulting in the frequent interruption of current paths, reduced conductive loss, and consequently limited absorption strength and bandwidth.

In addition, the lattice and electronic structure modifications induced by B doping have a profound influence on the dielectric loss mechanism. Although the catalytic growth process reduces the density of stacking faults (SFs), thereby diminishing defect-polarization-related relaxation and adversely affecting absorption performance, the effects arising from B incorporation into the lattice largely compensate for—and even outweigh—this drawback. Specifically, substitutional B atoms replacing Si in the SiC lattice act as acceptors, introducing shallow energy levels and significantly increasing hole concentration, thereby enhancing the intrinsic electrical conductivity of the material and boosting conductive loss. Furthermore, the local lattice distortion caused by doping, along with the formation of point defects or defect complexes, can generate stable electric dipoles that contribute additional defect-polarization loss within the X-band. Meanwhile, B doping alters the conductivity distribution at the nanowire–matrix interfaces, reduces interfacial potential barriers, and in conjunction with the increased surface area resulting from the higher aspect ratio, markedly strengthens Maxwell–Wagner–Sillars interfacial polarization. The combined enhancement of conductive and relaxation losses within the relevant frequency range enables the B-doped sample to achieve superior electromagnetic wave dissipation over a broader bandwidth, whereas the undoped sample, constrained by its morphology, insufficient conductive network, and underdeveloped polarization processes, exhibits a single dielectric-loss mechanism, leading to noticeably lower *RL* and *EAB*.

**Figure 8:**
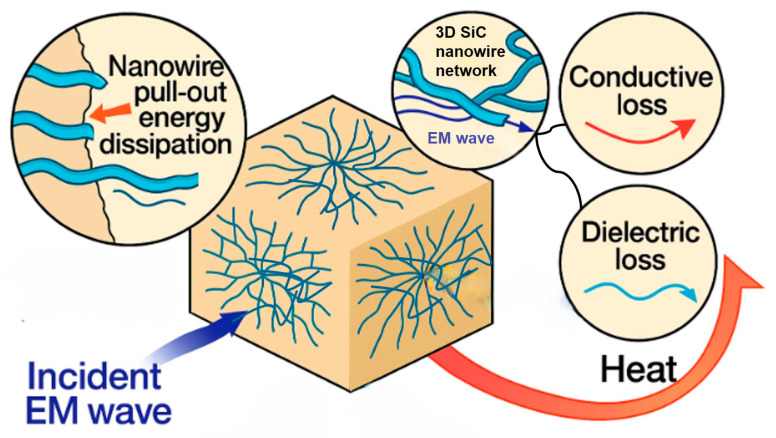
Schematic illustration of the mechanical-reinforcement and microwave-absorption mechanisms in the SiC nanowire/silicone composite.

Figure 8 illustrates the mechanisms by which the composite is mechanically reinforced and absorbs electromagnetic waves. Under external loading, the SiC nanowires embedded in the silicone matrix are the first to bear the applied stress and effectively pin sharp cracks to their vicinity. The crack front is forced to deflect, bend, or even branch along the nanowire–matrix interfaces, greatly lengthening its propagation path. As the load increases, interfacial debonding progresses and each nanowire undergoes sequential stretching, interfacial sliding, and eventual pull-out between the opposing fracture surfaces. Friction at the interface together with elastic-plastic deformation of the nanowire converts a substantial portion of the mechanical energy into heat, thereby markedly enhancing both the tensile strength and fracture toughness of the composite.

When electromagnetic waves impinge on the material, charge carriers driven by the microwave field oscillate within the SiC nanowire 3D conductive network and dissipate energy as Joule heat, giving rise to conduction loss. Simultaneously, the pronounced contrast in conductivity and polarizability between the semiconducting nanowires and the insulating silicone causes Maxwell–Wagner interfacial polarization. The build-up and relaxation of this polarization provides an additional dielectric-loss channel that further converts electromagnetic energy into heat. Repeated scattering and reflection of the waves within the three-dimensional network elongate the propagation path, allowing conduction and dielectric losses to compound and ultimately yielding broadband, high-efficiency microwave absorption.

As summarized in Table 1, our B-doped SiC nanowire/silicone rubber composite achieves a minimum *RL* of ≈ −35 dB and an *EAB* of ≈ 4.2 GHz at only 5 wt% and 3.5–4.0 mm. Compared with PPy-coated CNF/silicone rubber (4.0 mm, −25 dB, X-band coverage) [9], our system shows much deeper loss with thinner loading sections. Relative to NPC@MoSe_2_/PDMS, which needs 15 wt% loading to reach −51.6 dB with a 7.1 GHz bandwidth at 2.6 mm [39], we achieve a comparable absorption depth at a significantly lower filler ratio, albeit with narrower bandwidth. Similarly, MoO_2_/N-doped carbon nanowires in TPU require 40 wt% loading to approach −35 dB with a 3.26 GHz bandwidth at 2.3 mm [10], while our design delivers the same depth at ~8× lower loading. The rGO/TiO_2_-ODA/PDMS fabric reaches −47.4 dB with 7.7 GHz *EAB* at 3.5–4.5 mm [40], but depends on highly conductive graphene-based frameworks, in contrast to our all-dielectric system. Ti-HEO/ACET (17.5 wt% ACET) displays the strongest absorption (−52.3 dB) and the broadest bandwidth (6.12 GHz at ~2 mm) [12], but relies on compositional complexity and high-entropy oxides. The Fe–TiN fiber/PDMS system shows −20 dB at 1.8 mm with 12.2 GHz *EAB*, emphasizing high-temperature adaptability [11]. At the same 5 wt% loading, CNT-impregnated polyester nonwoven shows only −14.06 dB and 0.46 GHz [41], much weaker than our −35 dB and 4.2 GHz. Overall, our B-doped SiC nanowire network underscores superior filler efficiency, delivering strong absorption and multi-GHz bandwidth in a simple binary dielectric system at minimal filler content. The proposed fabrication route is industry-compatible and scalable. Boron-assisted growth enables large-scale synthesis of SiC nanowires with controlled defect states, forming an efficient 3D network at just 5 wt% when incorporated into silicone rubber. Although the high loading capacity of the nanowires increases the viscosity of the silicone rubber mixture, our process allows uniform dispersion and homogeneous microstructure through conventional industrial mixing and molding techniques. This compatibility facilitates integration into existing production lines without requiring specialized equipment.

The optimized thickness of 3.5–4 mm closely matches the quarter-wavelength condition in the X-band, enabling thin, conformal absorber layers for integration on metallic or curved surfaces without excessive weight or volume. Mechanically, the nanowire bridging effect enhances tensile strength (~5.7 MPa at 5 wt%) while maintaining high elongation, ensuring durability under bending, vibration, and thermal cycling. The all-dielectric, nonmagnetic composition prevents corrosion and magnetic interference issues, making the material suitable for harsh service environments. In summary, the combination of low filler content, low-cost raw materials, robust processing even under high-viscosity conditions, and scalability positions this system as a practical candidate for industrial implementation in lightweight and flexible microwave absorber architectures.

## 4. Conclusions

B-doped SiC nanowires were synthesized by microwave-assisted carbothermal reduction at 1400 °C for 30 min using H_3_BO_3_ as a catalytic dopant. Without boric acid, the nanowires grew via the classical vapor–solid (VS) mechanism. The introduction of H_3_BO_3_ led to its decomposition into B_2_O_3_, which reacted with SiO to form a low-melting B_2_O_3_–SiO liquid phase; this switched the growth to a vapor–liquid–solid (VLS) route. As a catalyst, boric acid accelerated mass transport, lowered the growth temperature, reduced stacking fault density, and promoted the formation of straighter nanowires with a higher aspect ratio, thereby facilitating the construction of a continuous 3D conductive network in the composite. Simultaneously, as a dopant, B atoms were substitutionally incorporated into the SiC lattice, enhancing crystallinity, increasing carrier concentration, and introducing defect and interfacial polarization centers. These changes jointly improved both the conductive and relaxation loss mechanisms, leading to significantly enhanced microwave absorption performance. Incorporated into a silicone matrix, the nanowires yielded composites with high mechanical strength (5.7 MPa) and strong microwave absorption (−35 dB, 4.2 GHz bandwidth at 3.5 mm) at 5 wt% loading, while higher loading (10 wt%) caused agglomeration and performance loss.

This synthesis method offers a short reaction time, high yield, and simple processing with readily available raw materials, making it scalable for industrial production. The dual role of boric acid in catalyzing nanowire growth and simultaneously tuning dielectric loss mechanisms, combined with the mechanical robustness and tunable microwave absorption, makes these composites promising for lightweight aerospace panels and protective housings for high-frequency electronics.

## Figures and Tables

**Table 1:** Comparison of the electromagnetic wave absorption performance of this work with representative flexible absorber systems.

Material System	Filler Loading (wt%)	Thickness (mm)	*RL*_min_ (dB)	*EAB* (GHz)
PPy-coated CNF/PDMS [9]	-	4.0	−25	4.2
NPC@MoSe_2_/PDMS [39]	15	2.6	−51.6	7.1
MoO_2_/N-doped carbon nanowire/TPU [10]	40	2.3	−35	3.26
rGO/TiO_2_–ODA/PDMS [40]	6.9	3.5	−47.4	7.7
Ti–HEO/ACET [12]	17.5 (ACET)	2.03	−52.31	6.12
Fe–TiN fiber/PDMS [11]	15	1.8	−20	12.2
CNT-impregnated polyester nonwoven (PU binder) [41]	5	1.0	−14.06	0.46
B-doped SiC nanowire/silicone (This work)	5	3.5–4.0	−35	4.2

## Data Availability

The original contributions presented in this study are included in the article. Further inquiries can be directed to the corresponding author(s).

## References

[1] Cheng Y., Seow J.Z.Y., Zhao H., Xu Z.J., Ji G. (2020). A Flexible and Lightweight Biomass-Reinforced Microwave Absorber. Nano-Micro Lett..

[2] Zou Y., Huang X., Fan B., Tang Z., Zhou J., Peng P., Liu X., Zhu J., Liu Y., Yue J. (2022). Constructing hierarchical Ti_3_SiC_2_ layer and carbon nanotubes on SiC fibers for enhanced electromagnetic wave absorption. Ceram. Int..

[3] Liu Y., He K., Chen G., Leow W.R., Chen X. (2017). Nature-Inspired Structural Materials for Flexible Electronic Devices. Chem. Rev..

[4] Mao L., Meng Q., Ahmad A., Wei Z. (2017). Mechanical Analyses and Structural Design Requirements for Flexible Energy Storage Devices. Adv. Energy Mater..

[5] Zhou Y., Sun Z., Jiang L., Chen S., Ma J., Zhou F. (2020). Flexible and conductive meta-aramid fiber paper with high thermal and chemical stability for electromagnetic interference shielding. Appl. Surf. Sci..

[6] Sheng M., Yang R., Gong H., Zhang Y., Lin X., Jing J. (2022). Enhanced thermal conductivity and stability of boron nitride/phenyl silicone rubber composites via surface modification and grain alignment. J. Mater. Sci..

[7] Ji J., Ge X., Pang X., Liu R., Wen S., Sun J., Liang W., Ge J., Chen X. (2019). Synthesis and Characterization of Room Temperature Vulcanized Silicone Rubber Using Methoxyl-Capped MQ Silicone Resin as Self-Reinforced Cross-Linker. Polymers.

[8] Gnanaseelan M., Trommer K., Gude M., Stanik R., Przybyszewski B., Kozera R., Boczkowska A. (2021). Effect of Strain on Heating Characteristics of Silicone/CNT Composites. Materials.

[9] Shi Y., Yu L., Li K., Li S., Dong Y., Zhu Y., Fu Y., Meng F. (2020). Well-matched impedance of polypyrrole-loaded cotton non-woven fabric/polydimethylsiloxane composite for extraordinary microwave absorption. Compos. Sci. Technol..

[10] Zhang X., Gong M., Dai Y., Wen B. (2022). Construction of one-dimensional MoO_2_/NC heteronanowires for microwave absorption. RSC Adv..

[11] Li C., Li D., Zhang L., Zhang Y., Zhang L., Gong C., Zhang J. (2022). Boosted microwave absorption performance of transition metal doped TiN fibers at elevated temperature. Nano Res..

[12] Li Y., Jin Y., Raza H., Wang Y., Chen Q., Zou X., Ren Z., Guo J., Zheng G., Cheng J. (2025). Dual driving strategy from micro-polarization to macroscopic conductance: Tailoring optimized low-frequency and wide-band microwave absorption in high-entropy oxides. J. Mater. Sci. Technol..

[13] Luo H., Chen W., Zhou W., Long L., Deng L., Xiao P., Li Y. (2017). Carbon fiber/Si3N4 composites with SiC nanofiber interphase for enhanced microwave absorption properties. Ceram. Int..

[14] Wu R., Zhou K., Yang Z., Qian X., Wei J., Liu L., Huang Y., Kong L., Wang L. (2012). Molten-salt-mediated synthesis of SiC nanowires for microwave absorption applications. CrystEngComm.

[15] Kuang J., Jiang P., Ran F., Cao W. (2016). Conductivity-dependent dielectric properties and microwave absorption of Al-doped SiC whiskers. J. Alloys Compd..

[16] Kuang J., Qin Q., Xiao T., Hou X., Jiang P., Wang Q., Cao W. (2019). Tunable dielectric permittivity and microwave absorption properties of Pt-decorated SiC nanowires prepared by magnetic sputtering. Mater. Lett..

[17] Kuang J., Xiao T., Zheng Q., Xiong S., Wang Q., Jiang P., Liu W., Cao W. (2020). Dielectric permittivity and microwave absorption properties of transition metal Ni and Mn doped SiC nanowires. Ceram. Int..

[18] Liu H., Zhang X., Xu N., Han C., Wu N., Wang B., Wang Y. (2024). Progress of One-Dimensional SiC Nanomaterials: Design, Fabrication and Sensing Applications. Nanomaterials.

[19] Xiao S., Mei H., Han D., Cheng L. (2019). 3D printed SiC nanowire reinforced composites for broadband electromagnetic absorption. Ceram. Int..

[20] Xu X., Wang D., Rao Z., Wu J., Zhou Y. (2021). In-situ Synthesis and Oxidation Resistance of Sialon/SiC Composite Ceramics Applied as Solar Absorber. J. Wuhan Univ. Technol. Sci. Ed..

[21] Shi R., Liu Z., Liu W., Kuang J. (2023). Preparation and Electromagnetic Wave Absorption Properties of N-Doped SiC Nanowires. Materials.

[22] Sun Z.G., Wang S.J., Qiao X.J., Li Y., Zheng W.H., Bai P.Y. (2018). Synthesis and microwave absorbing properties of SiC nanowires. Appl. Phys. A.

[23] Zhang D., Hao Z., Qian Y., Zeng B., Zhu H., Wu Q., Yan C., Chen M. (2018). The design and performance of the nano-carbon based double layers flexible coating for tunable and high-efficiency microwave absorption. Appl. Phys. A.

[24] Kuang J., Cao W., Jacobson N. (2014). Oxidation Behavior of SiC Whiskers at 600–1400 °C in Air. J. Am. Ceram. Soc..

[25] Zhang Y., Yin X., Ye F., Kong L. (2014). Effects of multi-walled carbon nanotubes on the crystallization behavior of PDCs-SiBCN and their improved dielectric and EM absorbing properties. J. Eur. Ceram. Soc..

[26] Qiao L., Han X., Gao B., Wang J., Wen F., Li F. (2009). Microwave absorption properties of the hierarchically branched Ni nanowire composites. J. Appl. Phys..

[27] Lu M.-M., Cao W.-Q., Shi H.-L., Fang X.-Y., Yang J., Hou Z.-L., Jin H.-B., Wang W.-Z., Yuan J., Cao M.-S. (2014). Multi-wall carbon nanotubes decorated with ZnO nanocrystals: Mild solution-process synthesis and highly efficient microwave absorption properties at elevated temperature. J. Mater. Chem. A.

[28] Duan W., Yin X., Cao F., Jia Y., Xie Y., Greil P., Travitzky N. (2015). Absorption properties of twinned SiC nanowires reinforced Si_3_N_4_ composites fabricated by 3D-prining. Mater. Lett..

[29] Hao Y.-J., Wagner J.B., Su D.S., Jin G.-Q., Guo X.-Y. (2006). Beaded silicon carbide nanochains via carbothermal reduction of carbonaceous silica xerogel. Nanotechnology.

[30] Chang B., Gersten B.L., Szewczyk S.T., Adams J.W. Towards the Preparation of Boron Carbide Nanorods by Carbothermal Reaction Method. Proceedings of the Technical Proceedings of the 2006 NSTI Nanotechnology Conference and Trade Show, Volume 1.

[31] Luo X., Ma W., Zhou Y., Liu D., Yang B., Dai Y. (2009). Synthesis and Photoluminescence Property of Silicon Carbide Nanowires Via Carbothermic Reduction of Silica. Nanoscale Res. Lett..

[32] Soltys L., Mironyuk I., Mykytyn I., Hnylytsia I., Turovska L. (2023). Synthesis and Properties of Silicon Carbide (Review). Phys. Chem. Solid State.

[33] Yang Q., Wang H., Lei R., Xu S. (2017). Low temperature formation of AlN nanofibers by carbothermal reduction nitridation of hydrothermal precursor fibers. J. Asian Ceram. Soc..

[34] Zhang Y., Xu J., Feng Y., Qiu T. (2018). Hot-air aging failure mechanisms of carbonyl iron powder/methyl vinyl silicone rubber microwave-absorbing materials. Adv. Polym. Technol..

[35] Yan X., Guo J., Jiang X. (2022). The microwave-absorption properties and mechanism of phenyl silicone rubber/CIPs/graphene composites after thermal-aging in an elevated temperature. Sci. Rep..

[36] Wang H., Wu L., Jiao J., Zhou J., Xu Y., Zhang H., Jiang Z., Shen B., Wang Z. (2015). Covalent interaction enhanced electromagnetic wave absorption in SiC/Co hybrid nanowires. J. Mater. Chem. A.

[37] Ji S., Zhang S., Wang Z., Li C., Cao W., Zhu Y., He C., Chen Y. (2023). High-Impact Performance and Thermal Properties of Polyimine Nanocomposites Reinforced by Silicon Carbide Nano-Whiskers. Materials.

[38] Chi Q., Fang H., Meng Z., Zhang C., Li Z., Tang C., Zhang T. (2022). Improved electrical, thermal, and mechanical properties of silicone rubber-based composite dielectrics by introducing one-dimensional SiC fillers. J. Mater. Sci. Mater. Electron..

[39] Zhang Y., Zhang L., Zhou B., Ahmad M., Zhang Q., Zhang B. (2023). Microwave absorption and thermal conductivity properties in NPC@MoSe_2_/PDMS composites. Carbon.

[40] Zhang Z., Meng Y., Fang X., Wang Q., Wang X., Niu H., Zhou H. (2024). Robust, Flexible, and Superhydrophobic Fabrics for High-Efficiency and Ultrawide-Band Microwave Absorption. Engineering.

[41] Gu W., Shi J., Zhang J., Jia Q., Liu C., Ge H., Sun Q., Zhu L. (2023). Fabrication and Investigation of the Microwave Absorption of Nonwovens Modified by Carbon Nanotubes and Graphene Flakes. Molecules.

